# Longitudinal HbA_1c_ trajectory modelling reveals the association of HbA_1c_ and risk of hospitalization for heart failure for patients with type 2 diabetes mellitus

**DOI:** 10.1371/journal.pone.0275610

**Published:** 2023-01-20

**Authors:** Clarence Tee, Haiyan Xu, Xiuju Fu, Di Cui, Tazeen H. Jafar, Yong Mong Bee

**Affiliations:** 1 Systems Science Department, Institute of High-Performance Computing, Singapore, Singapore; 2 Department of Advanced Design and Systmes Engineering, City University of Hong Kong, Kowloon Tong, Hong Kong; 3 Duke-NUS Medical School, National University of Singapore, Singapore, Singapore; 4 Department of Endocrinology, Singapore General Hospital, Singapore, Singapore; University of Dundee, UNITED KINGDOM

## Abstract

**Background:**

Inconsistent conclusions in past studies on the association between poor glycaemic control and the risk of hospitalization for heart failure (HHF) have been reported largely due to the analysis of non-trajectory-based HbA_1c_ values. Trajectory analysis can incorporate the effects of HbA_1c_ variability across time, which may better elucidate its association with macrovascular complications. Furthermore, studies analysing the relationship between HbA_1c_ trajectories from diabetes diagnosis and the occurrence of HHF are scarce.

**Methods:**

This is a prospective cohort study of the SingHealth Diabetes Registry (SDR). 17,389 patients diagnosed with type 2 diabetes mellitus (T2DM) from 2013 to 2016 with clinical records extending to the end of 2019 were included in the latent class growth analysis to extract longitudinal HbA_1c_ trajectories. Association between HbA_1c_ trajectories and risk of first known HHF is quantified with the Cox Proportional Hazards (PH) model.

**Results:**

5 distinct HbA_1c_ trajectories were identified as 1. low stable (36.1%), 2. elevated stable (40.4%), 3. high decreasing (3.5%), 4. high with a sharp decline (10.8%), and 5. moderate decreasing (9.2%) over the study period of 7 years. Poorly controlled HbA_1c_ trajectories (Classes 3, 4, and 5) are associated with a higher risk of HHF. Using the diabetes diagnosis time instead of a commonly used pre-defined study start time or time from recruitment has an impact on HbA_1c_ clustering results.

**Conclusions:**

Findings suggest that tracking the evolution of HbA_1c_ with time has its importance in assessing the HHF risk of T2DM patients, and T2DM diagnosis time as a baseline is strongly recommended in HbA_1c_ trajectory modelling. To the authors’ knowledge, this is the first study to identify an association between HbA_1c_ trajectories and HHF occurrence from diabetes diagnosis time.

## Introduction

The relation between heart failure and type 2 diabetes mellitus (T2DM) has been extensively studied, with the vast majority coming to the consensus that those diagnosed with T2DM are at elevated risk of heart failure [1, 2]. The driving force behind the pathogenesis of T2DM-induced heart failure remains unclear, as acknowledged by recent reviews and studies, even though several mechanisms have been postulated [3]. Furthermore, certain glucose-lowering therapies have attributed to an increased risk of heart failure, as evidenced by clinical trials on certain medications such as thiazolidinediones and dipeptidyl peptidase-4 (DPP-4) inhibitors, notably the Saxagliptin Assessment of Vascular Outcomes Recorded in Patients with Diabetes Mellitus (SAVOR)-Thrombolysis in Myocardial Infarction (TIMI) 53 trial, adding another dimension of complexity to the treatment of T2DM [4] since glycaemic control is still prioritised as primary treatment for T2DM patients.

In randomized trials or large-scale studies, glycated haemoglobin (HbA1c) levels are often averaged or taken at a few discrete points over a period of follow-up. As such, improved glycaemic control via reduced HbA_1c_ levels is to have little to no significance in minimizing the risk of macrovascular complications [5]. These results have prompted questions about the validity of the role averaged HbA_1c_ measures have on cardiovascular events, suggesting glycaemic variability as a preferred measure [6]. Recognition of the lack of information from standalone or averaged HbA_1c_ values has also led to the search for innovative ways to model and analyse glycaemic control over time for prolonged periods. However, such methods face issues on interpretability [7]. Extracting trajectories from a cohort gives individual subjects insight on the progression of their condition and potential generalized outcomes, ultimately providing motivation for greater self-management based on observable trends apparent in the larger population. The advancement towards personalized treatment is also timely, where standardized approaches to treating T2DM and preventing complications are evidenced to be no longer effective [8].

A systematic review of longitudinal studies on HbA_1c_ trends indicated that most of pre-existing studies used the time from recruitment instead of diabetes diagnosis time as an independent variable and these studies focused primarily on microvascular outcomes and/or mortality [9, 10]. In other studies, HbA_1c_ values were measured once a year which neglects the potential effects of glycaemic variability on outcomes brought about by more frequent measurements [10, 11]. Other studies modelled HbA1c trajectories via a linear relation to time with Latent Class Growth Analysis (LCGA) across a number of years after diabetes diagnosis. Although a prevalent association to microvascular outcomes was found, it was limited to the follow-up period [11, 12]. Hence, such a sequential approach to trajectory analysis, whereby trajectories are extracted before drawing associations to outcomes, is inefficient and expensive which hinders early interventions. For example, the knowledge of the HbA1c trajectory starting from the point of T2DM diagnosis may prompt physicians to select treatment more targeted at heart failure prevention (such as SGLT2 inhibitors) versus just taking into consideration the latest HbA1c.

This paper aims to extract trajectories of HbA_1c_ to identify subgroups to study the association between the HbA_1c_ trajectories and the risk of heart failure right from the time of diabetes diagnosis, not missing out on the association presented at the early diabetes stage. The subgroups share a degree of homogeneity regarding their T2DM progression via HbA_1c_ trends without being constrained to a linear trend.

## Material and methods

### Data source, study population and study design

The SingHealth Diabetes Registry (SDR) is a database holding patient information ranging from socio-economic demographics to laboratory test results which have been collected from public health institutions [13]. The registry contains electronic health record data collected from T2DM patients who visited any SingHealth institution for treatment purposes. More specifically, the SDR recorded casemix variables and outcome variables. Casemix variables include demographic (birth date, gender, ethnicity, etc) and lifestyle factors, diagnosis profile, treatment factors and anthropometric variables while outcome variables include laboratory results, clinical episodes, surgical procedures and vaccinations. We have obtained written consent from SingHealth Centralised Institutional Review Board (CIRB Ref: 2019/2414) and A*STAR Institutional Review Board (IRB Ref: 2019–079) to waive further ethical deliberation because this application involves analysis of anonymised datasets from the SDR at a sandbox environment in the Health Services Research Centre.

This study is a longitudinal prospective cohort study from 2013 to 2019 with a focus on subjects diagnosed with T2DM from 2013 to 2016. Out of a total 20,794 subjects, each subject is required to have at least 5 HbA_1c_ samples across the span of at least 3 years, which yields a final sample size of 17,389 (100%) subjects. It was necessary to define a common baseline shared across all subjects, which provides a basis for a fair comparison instead of using the first recorded HbA_1c_ specimen date of each subject as a baseline. This step comes as there are subjects whose year of T2DM diagnosis differs from the year when the first HbA_1c_ specimen was taken. If the year of the first recorded HbA_1c_ specimen is the same as the year of T2DM diagnosis, the Diabetes Diagnosis Date (DDD) would be assumed to be the date of the first recorded HbA_1c_ specimen. For subjects with a mismatch between their year of T2DM diagnosis and the year of their first recorded HbA_1c_ specimen, DDD is set as a random variable drawn from a uniform distribution between 0 and 365 in the year of T2DM diagnosis. The total number of subjects who require random imputation of DDD is 3,248 (19%), which is still a sizable percentage to be removed from the analysis completely. Hence random imputation is preferred over dropping these subjects. The event of interest for this analysis is defined as the onset of heart failure where hospitalization was required. With an estimated DDD as a starting point, the first HHF after DDD is identified and only HHF as a primary diagnosis on discharge is accepted as an event (ICD-10 codes I11, I13, and I50).

### Statistical methods

Latent class growth analysis (LCGA), a model-based clustering technique, is used in this study to cluster HbA_1c_ trajectories, which falls under the category of unsupervised learning and belongs to a broader family of models known as longitudinal Finite Mixture Modelling (FMM) [14]. Within this cohort of patients, it is assumed that multiple latent classes of patients sharing a degree of similarity in their HbA_1c_ trajectories exist in the data and it is desired to minimize within cluster heterogeneity while simultaneously maximizing heterogeneity between clusters.

The R package “lcmm” [15] was used to model and cluster HbA_1c_ trajectories to obtain probabilities of class membership for each subject. A linear, quadratic, cubic, logarithmic, and exponential relation between HbA_1c_ and the number of weeks from baseline was modelled and up to and including 10 latent classes were run (1 latent class implies no clustering was done at all) for each modelled relation. A grid search method was implemented to ensure a global maximum was found by generating 20 random sets of initial values.

For each model specification, the optimal number of latent classes was determined by two conditions: the Bayesian Information Criterion (BIC) [16] and the lowest proportion of subjects in a class. The proportion for each class was set at a threshold of 3%, given the large sample size used in this study [17]. A 10-fold cross-validation procedure [18] was applied to further validate model fit. The Cox PH model is subsequently used to quantify the associations between extracted trajectories and HHF after adjusting for demographic characteristics and known prior microvascular and macrovascular complications.

Based on the extracted HbA_1c_ trajectories, it was discovered that a particular trajectory falls below another with distinctly different characteristics sometime after DDD. In order to study the significance of setting DDD as a baseline for HbA_1c_ extraction, a baseline other than the DDD was implemented and differences in class membership was subsequently compared. HHF risk of subjects who were re-grouped was further quantified by the Cox PH model. The predictive ability of latent classes on risk of HHF is demonstrated as well by splitting the cohort and predicting the posterior probability of class assignment for subjects in a testing set (20%) using parameter estimates from the training set (80%). The risk of HHF is subsequently predicted for the subjects in the validation set.

All analyses were carried out using R version 3.6.0.

## Results

### Model selection

Model selection results are summarized in Table 1. For each model, the data was fit with the increasing number of latent classes, and it was observed that BIC continually decreases with the increase in the number of classes. Hence, the minimum proportion of subjects clustered in a class has to be used to determine the optimal number of classes, which is reported in Table 1. From Table 1, with 6 latent classes across all model specifications, the minimal proportion of subjects falls below the threshold of 3% hence 5 latent classes are the optimal number of classes. A 10-fold cross-validation further confirmed the same results.

**Table 1:** Lowest proportion of subjects in a latent class for complete dataset.

Classes (#)	Linear	Quadratic	Cubic	Logarithmic	Exponential
2	0.141	0.142	0.144	0.152	0.141
3	0.074	0.073	0.072	0.073	0.074
4	0.036	0.038	0.052	0.07	0.036
**5**	**0.032**	**0.033**	**0.035**	**0.031**	**0.032**
6	0.025	0.029	0.029	0.025	0.024

Table 2 shows that the logarithmic model specification has the lowest BIC value amongst the linear, quadratic, cubic, and exponential model and hence the logarithmic model with five classes is selected as the final model. The scaled entropy [14] for the logarithmic model is 0.858, and it has been shown that BIC is adequate as a model selection criterion for entropy values larger than 0.8 [19].

**Table 2:** Summary of model fit on all subjects for 5 latent classes across all model specifications.

Model Specification	BIC Value
Logarithmic	790730.5
Linear	810598.1
Quadratic	805766.7
Cubic	801714.5
Exponential	812110.7

The average posterior probability of assignment (APPA) [14] is reported in Table 3 for the final model of 5 latent classes with a logarithmic model. It is the posterior probability of subjects clustered to class *k* given that they have been assigned to the *k*-th respective class on average, which is interpreted as a measure of certainty. APPA would ideally be greater than 0.7 and values close to 1 are measures which indicate a good model fit [14].

**Table 3:** Average posterior probability of the 5 latent class, logarithmic model specification.

	Class 1	Class 2	Class 3	Class 4	Class 5
**Average Posterior Probability**	0.914	0.87	0.977	0.916	0.948

### HbA_1c_ trajectories

**Fig 1:**
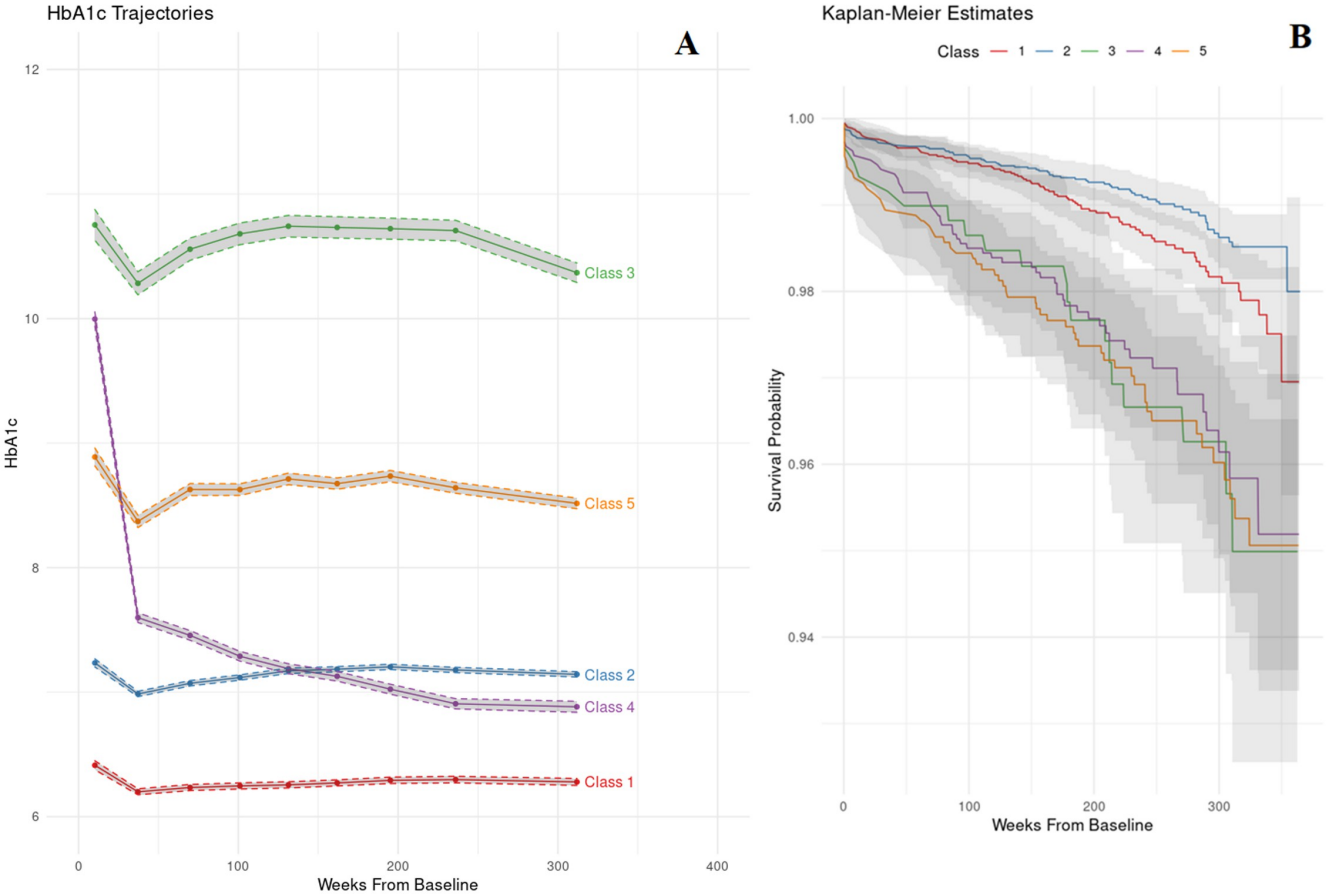
(A) HbA1c trajectories of the 5 classes. (B) Kaplan-Meier curves of the 5 classes.

Fig 1A visualizes the five trajectories extracted from the logarithmic model, which can be classified into subgroups of subjects with: low stable (35.5%, “Class 1”), moderate low stable (41.1%, “Class 2”), high decreasing (3.4%, “Class 3”), high with a sharp decline (10.7%, “Class 4”) and moderate high decreasing (9.2%, “Class 5”). All five classes experienced a drop in HbA_1c_ levels of varying magnitudes within the initial weeks of T2DM diagnosis. Class 1 has the lowest HbA_1c_ levels followed by Class 2. Class 3 has the highest HbA_1c_ levels. Class 4 starts with a very high HbA_1c_ level followed by a steep decline during the first approximate 30 weeks before transiting to a more gradual decline. The HbA_1c_ trajectory of subjects in Class 4 declines approximately to that of subjects in Class 2 at about 25 weeks from baseline. Class 5 has moderately high HbA_1c_ levels. Variability of HbA1c in Class 3,4 and 5 are distinctly larger than Class 1 and 2.

Table 4 shows the baseline characteristics of the subjects in all 5 classes. Subjects in Class 1 and 2 are older at the time of diagnosis. Subjects in Class 3 have the highest HbA_1c_ at baseline whereas those in Class 1 have the lowest. There is also a slight shift in the composition of ethnic demographics in Class 3, 4 and 5, with increased proportions of Malay subjects compared to Class 1 and 2.

**Table 4:** Baseline characteristics of the five classes.

	Class 1	Class 2	Class 3	Class 4	Class 5
N (%)	6,172 (35.5)	7,148 (41.1)	594 (3.4)	1,869 (10.7)	1,606 (9.2)
Mean age at diagnosis, years	61.6 ± 10.9	57.8 ± 10.7	49.2 ± 11.7	55.4 ± 10.5	53.2 ± 12.1
Male, %	49.0	51.7	54.2	57.6	53.5
Ethnicity, %					
Chinese	77.0	71.4	41.7	64.1	52.4
Malay	13.7	16.2	34.1	22.6	27.5
Indian	6.1	8.7	16.8	9.6	13.3
Other	3.2	3.7	7.4	3.7	6.8
Mean HbA_1c_ at diagnosis (SD), %	6.23 (0.59)	7.16 (0.86)	10.6 (2.14)	7.69 (1.81)	8.73 (1.7)
Mean HbA_1c_ Frequency (SD)	13.5 (4.6)	16.3 (5.5)	13.5 (7.6)	16.5 (5.5)	16.3 (7.0)
Mean observational period (SD), weeks	239.9 (65.6)	252.5 (67.3)	244.6 (75.1)	233.9 (68)	252.8 (72.2)
HHF observed, %	1.43	1.05	3.37	2.89	3.55
Established CVD, %	16.1	13.36	7.74	10.05	11.96
Prior IHD, %	9.61	8.24	5.05	5.94	8.03
Prior PAD, %	0.37	0.32	0.84	0.695	0.31
Prior Haemorrhagic Stroke, %	0.57	0.35	0	0.268	0.44
Prior Ischaemic Stroke, %	5.2	3.27	1.01	2.68	1.99
Prior TIA, %	1.34	0.89	0.67	0.43	0.81
Prior AF, %	1.38	0.99	0.51	1.07	0.934
Prior Neuropathy, %	0.34	0.364	1.35	1.02	0.498
Prior DPA, %	0.292	0.238	1.35	1.50	0.56
Prior Heart Failure, %	0.437	0.504	0.673	0.59	1.18

### Association between HbA1c trajectories and risk of HHF

Kaplan-Meier curves of the five classes are shown in Fig 1B. Subjects in classes 1 and 2 share similar event-free survival probabilities in approximately the first 125 weeks but increasingly diverge thereafter with Class 2 having higher survival probabilities. Class 4 is associated with poorer survival probabilities than Class 1 and 2 throughout the follow-up period. This is despite the HbA_1c_ levels of subjects in Class 4 being lower than those in Class 2 after approximately 150 weeks. A log-rank test indicated differences in survival between subjects in Class 4 and Class 2 (hazard ratio of 3.48 and p<0.01).

Table 5 summarises hazard ratios and their significance after adjusting for population characteristics (age at diagnosis, ethnicity, and gender), established cardiovascular disease (CVD), prior ischemic heart disease (IHD), prior peripheral arterial disease (PAD), prior ischaemic stroke (IS), prior haemorrhagic stroke (HS), prior transient ischaemic attack (TIA), prior atrial fibrillation (AF), neuropathy, diabetic peripheral angiopathy (DPA) and prior heart failure (HF). Of the 17,389 subjects, information on prior complications at baseline were missing for 2,259 subjects, but they were included in the Cox PH model nonetheless for the sake of completeness. All subsequent analyses were adjusted for population characteristics and prior complications at baseline.

**Table 5:** Summary of associations between HbA1c trajectories and HHF after adjusting for multiple risk factors.

	Hazard Ratio (95% CI)	p-value
Class 1 (Base)	1	-
Class 2	0.79 (0.6 to 1.1)	0.15
Class 3	4.1 (2.4 to 6.7)	<0.01
Class 4	2.75 (1.9 to 3.9)	<0.01
Class 5	2.8 (2.0 to 4.0)	<0.01
Female (Base)	1	-
Male	1.2 (0.94 to 1.5)	0.14
Chinese (Base)	1	-
Indian	1.5 (0.98 to 2.2)	0.06
Malay	1.82 (1.4 to 2.4)	<0.01
Other races	1.9 (1.2 to 3.2)	0.01
Age at diagnosis	1.05 (1.04 to 1.06)	<0.01
Baseline prior complications versus those without (Base)	1	-
Missing Prior Complications	1.5 (0.9 to 2.2)	0.06
With Established CVD	3.4 (2.0 to 5.8)	<0.01
Prior IHD	1.5 (0.9 to 2.5)	0.096
Prior PAD	1.2 (0.5 to 3.2)	0.7
Prior Stroke, Haemorrhagic	0.86 (0.2 to 3.6)	0.83
Prior Stroke, Ischaemic	0.6 (0.32 to 1.1)	0.09
Prior TIA	0.78 (0.3 to 2.0)	0.6
Prior AF	3.1 (2.04 to 4.7)	<0.01
Prior Neuropathy	2.5 (1.08 to 5.7)	0.03
Prior DPA	2.8 (1.2 to 6.2)	0.01
Prior Heart Failure	5.4 (3.5 to 8.3)	<0.01

Subjects in Class 3, 4 and 5 have an elevated risk profile as reflected in the hazard ratios when compared to Class 1. Compared to the Chinese ethnicity, all other ethnicities had higher hazard ratios though it should be noted that sample sizes for other races are relatively small. Of all the prior complications considered at baseline, subjects with established CVD, prior AF or prior HF were at significantly higher risk of HHF compared to those without.

Of the 5 distinct trajectories, Class 4 stands out. Class 4 has a similar baseline to Class 3, but it rapidly declines and even falls below the stable trajectory (Class 2). Analysing the proportion of subjects in each class with specific prior complications, which are associated with a higher risk of HHF in Table 4 at baseline, it is observed overall that subjects in Class 4 have a higher proportion of subjects with these pre-existing prior complications, specifically, established CVD, prior AF, prior neuropathy, prior DPA and prior HF.

### Significant role of diabetes diagnosis time

**Fig 2:**
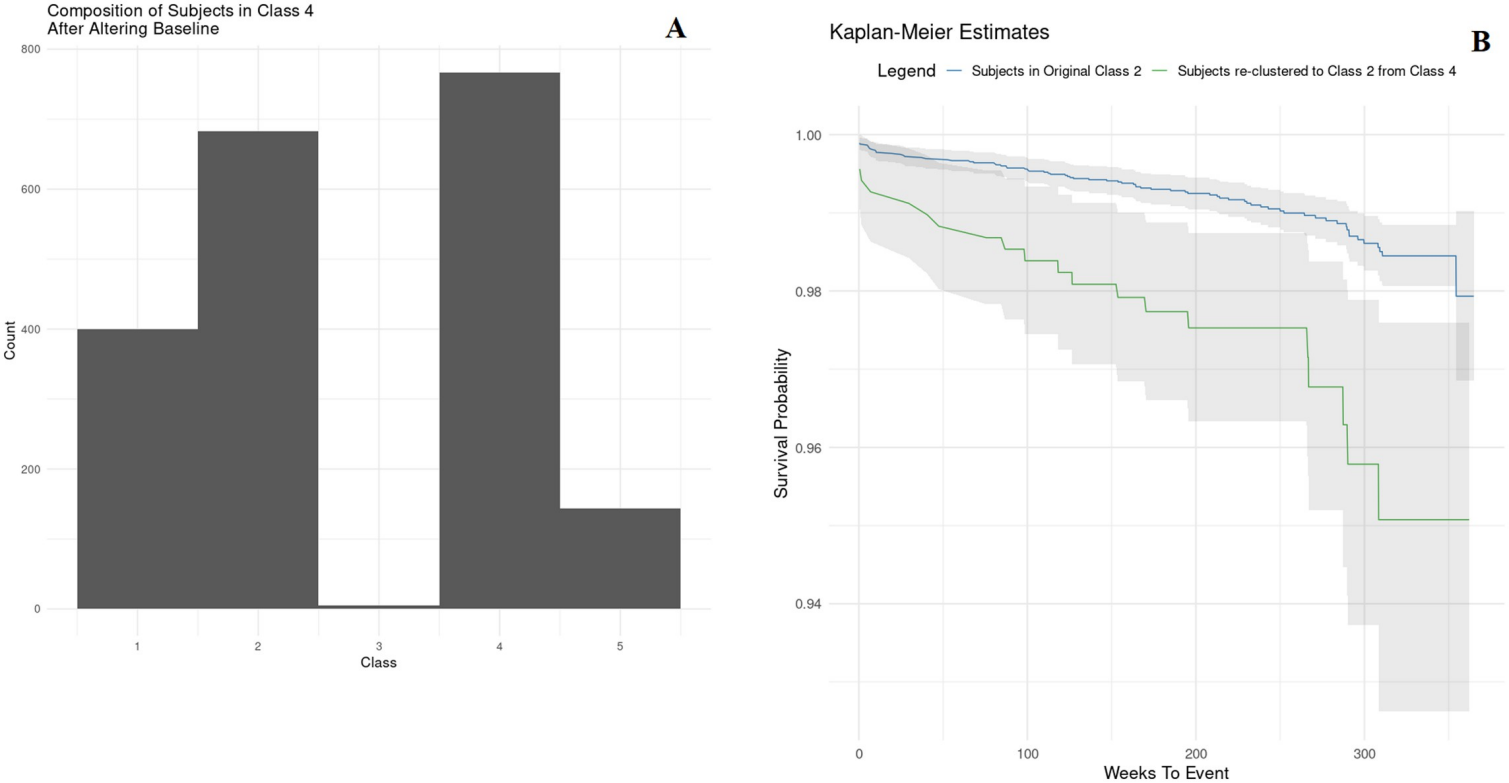
(A) Change in composition of subjects in the original Class 4 after altering baseline. (B) Survival curves of subjects in Class 2 after clustering from different baselines.

In Fig 2A, after re-grouping the patient in the original Class 4 with a new baseline defined as 50 weeks after the estimated DDD, only 38% of subjects retained their class membership after altering the baseline. 34% of subjects were re-grouped to Class 2 and 20% were re-grouped to Class 1. In Fig 2B, survival curves of subjects who were originally clustered to Class 2 and of subjects who were originally in Class 4 but had been re-grouped to Class 2 due to a new baseline are different. The latter group had a higher relative risk of HHF (HR = 2.1, 95% CI 1.19–3.7) with a p-value of 0.01, when a different baseline other than the estimated DDD was used. Adjusting for population characteristics and prior complications, the relative risk of HHF remains elevated (HR = 2.83, 95% CI 1.53–5.24) and p<0.01.

### Predictive ability of latent classes on HHF

To test the predictive ability of the latent class linear mixed model, the entire cohort of 17,389 (100%) subjects was split into a training and a test set. The optimal model was fitted on 13,911 subjects (≈80% of the cohort), and parameter estimates obtained were used to calculate the posterior probability of class memberships of the remaining 3,478 subjects (≈20% of the cohort). Classes were assigned to each subject in the validation set based on the highest calculated posterior probability of class membership and of the 3,478 subjects, approximately 94% retained their original class assignments. Separately thereafter, a multivariate Cox PH model considering all covariates in Table 5 as predictors of HHF, was fitted on the training set and predictions of risk scores were generated for the validation set. To determine the goodness-of-fit, a commonly used measure known as the concordance statistic [20] was computed to assess the model’s discriminative ability.

**Fig 3:**
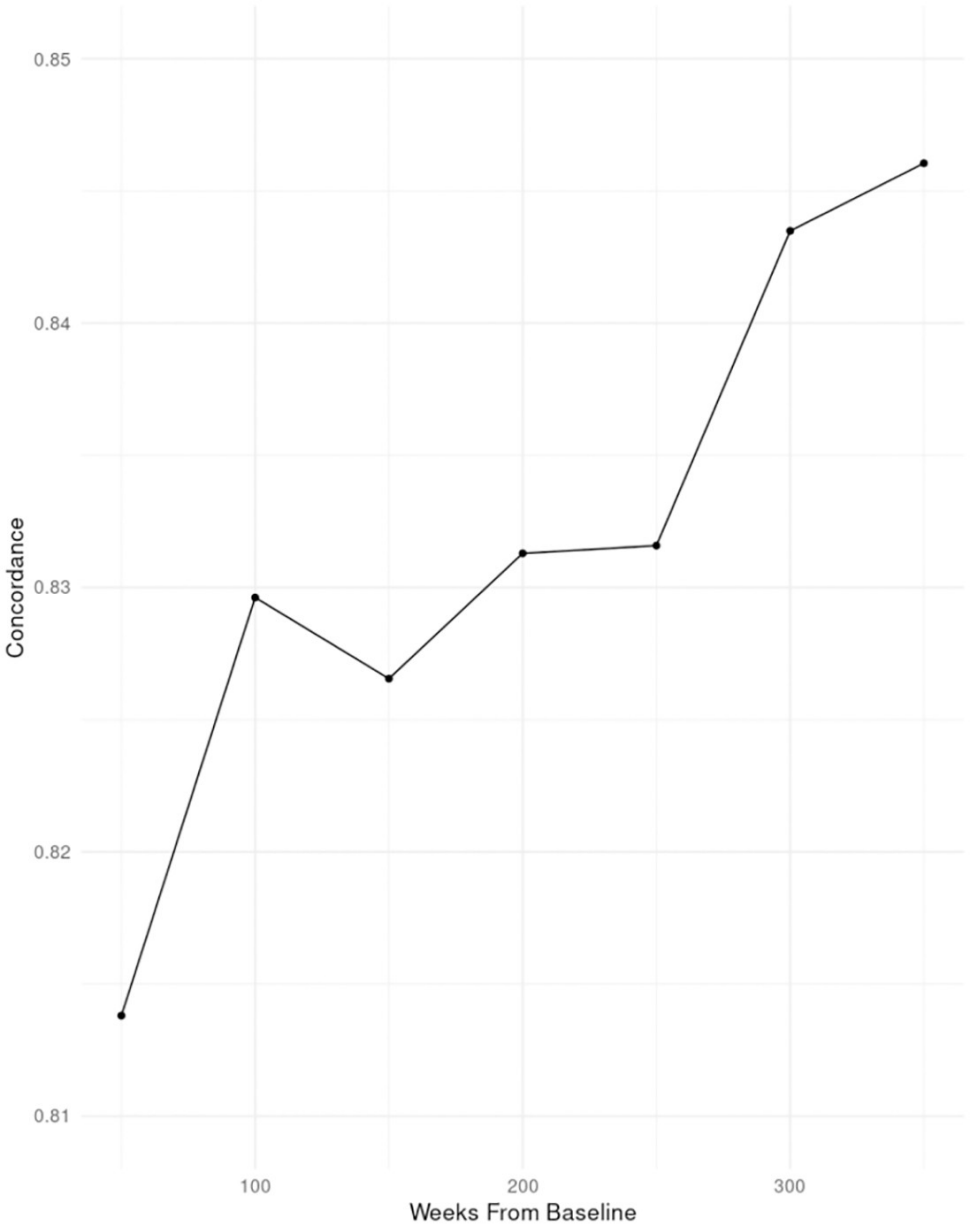
Increase in concordance statistic with more HbA_1c_ values of subjects on cumulative time intervals.

The predictive ability of latent classes on HHF is also evident across time since it is expected that more information on trajectorial values can converge to a more precise clustering of subjects with less uncertainty as time progresses. Within the validation set of 3,478 subjects, the sample was further divided into 7 disjoint time intervals, where each interval contained 50 weeks. After dividing the sample by time intervals, the sample size was reduced from 3,478 to 3,099 since a baseline had to be set at time 0. Trajectories of these subjects were restricted from time 0 to 50 weeks, where posterior probabilities of class memberships were computed based on parameter estimates from the training set. A multivariate Cox model fitted with the training set then predicted a risk score based on all covariates in Table 5, including latent class memberships as predictors of HHF, and a concordance index was obtained to reflect the goodness of fit for these subjects. This process was repeated iteratively with cumulative time intervals (for example, 0 to 100 weeks, 0 to 150 weeks etc.) and a trend of concordance with cumulative time intervals is plotted in Fig 3. A concordance statistic of 0.814 was achieved within the first 50 weeks, and it increased to 0.846 as more trajectorial HbA_1c_ values are included over the entire duration of 350 weeks, demonstrating the utility of tracking HbA1c trajectories over time.

We also obtained the concordance statistic of 0.76 for a simpler Cox PH model, which considers all covariates in Table 5 without the latent class memberships. It shows that the concordance statistic increases (0.846–0.76)/0.76 ≈11% when the latent class memberships are included.

## Discussion

In this study, the association between HbA_1c_ trajectories and HHF is reported, with results not reflected in previous studies. Low and stable HbA_1c_ trajectories are associated with a relatively lower risk of HHF. High mean HbA_1c_ levels have been reported to be associated with an increased risk of HHF [21–24], and the association between greater glycaemic variability and an increased risk of HHF is consistent with previous findings in [25–27]. HbA1c trajectories can provide more information than single HbA1c values. The HbA1c trajectories consist of both HbA1c value and its changes along the time elapsed since diabetes diagnosis. The changes along the time do provide more information, which has been demonstrated through the two points addressed in this study. One is that the HbA1c level of some patients in Class 4 after 50 weeks looks similar to the HbA1c level of patients in Class 2. However, the risk of HHF of subjects who were originally clustered to Class 2 and of subjects who were originally in Class 4 but had been re-grouped to Class 2 due to a new baseline are different. The other one is that predictability on the risk of HHF increases as longer HbA1c trajectories over the time were included. From these observations, analysing HbA_1c_ trajectories and its association to HHF has its advantages since trend patterns have been considered. Initial values of HbA_1c_ are inadequate in determining the risk of potential future HHF and using averaged HbA_1c_ values within a specified observation time window as most cross-sectional studies do, is of minimal value in determining the future risk of HHF.

For subjects with a mismatch between the year of diabetes diagnosis and the year of first recorded HbA_1c_ specimen, randomness was injected into the determination of DDD. To check if the randomness could affect the identification of trajectories, another set of DDD with a different random seed was generated and compared with the current results. Of the 17,389 subjects, 17,254 (99.22%) subjects retained their class membership when a different set of randomly drawn DDD was used as baseline. This is further quantified by a log-rank test comparing the survival curves of subjects across all 5 classes between the 2 randomly determined baselines. The Chi-Square test statistic returned 0 (p = 1) for all classes, thus concluding that the set of randomly seeded DDD almost yielded identical results.

With an estimated DDD as a baseline, compared to setting the date on which the first recorded HbA_1c_ specimen value was taken as a baseline can significantly affect results. A different relative risk of HHF was observed when the baseline was changed. Using the latter approach risks losing information on trajectories of subjects which would have otherwise been clustered into another group. This finding is consistent with a previous study that also associated with macrovascular risk with diabetes duration at T2DM diagnosis [28] which modelled a linear effect of diabetes duration on risk estimates. The use of an estimated DDD includes diabetes duration in this study without having to model it as a linear effect. Separately, the trajectory model was fitted to subjects who did not require random imputation of the DDD and 95.2% of these subjects retained their original class assignment. This method of random imputation does not drastically alter the stability of results, and it allows for more subjects to be included in the cohort which improves the generalizability of the model.

Class based HbA1c trajectories based on individuals’ trajectories have their merits. A class based HbA1c trajectory represents a subpopulation where the HbA1c trajectories share a similar trend. Associating longitudinal trajectories of biomarkers, such as HbA_1c_ to a specific outcome provides continuous, real-time tracking of class specific subjects on future risk of cardiovascular events. It demonstrates the possibility of predicting future events for subjects based on their historical trajectories (not only limited to HbA_1c_ trajectories) and other relevant status, which may potentially prompt early intervention, representing a step towards personalized treatment. The current study allows for the analysis of concurrent associations between HbA_1c_ trajectories and risk of HHF. Retrospective studies of HbA_1c_ trajectories and complications are prone to sources of error which proves difficult to track and control. These sources of error include confounding and bias, in turn affecting any and all estimates of relative risk, making the interpretation of results difficult for clinicians to obtain any actionable insights [29].

Class 4 (high with a sharp decline class) is unique considering that its baseline value is similar to Class 3 (high decreasing class), but it rapidly drops and coalesces with Class 2 (moderate low stable) when time evolves. With further study, we observed that patients in Class 4 had the highest metformin prescription rate within the first 20 weeks after diabetes was diagnosed among all groups. In detail, the rate across Class 1 to Class 5 is 25.1%, 46.2%, 74.2%, 81.8%, and 65.6%, respectively. It could be a possible reason to explain Class 4’s rapid drop. On the other hand, the HHF risk of Class 4 is similar to Class 3 but different from Class 2. A possible reason is that subjects in Class 4 have a relatively high proportion of subjects with pre-existing complications, specifically, established CVD, prior AF, and prior HF (see Table 4), which are associated with a significantly higher risk of HHF according to Table 5.

Limitations in this study do exist. The observational period of this study is only for 7 years. Since more data is being collected on a rolling basis, this study sets a foundation which allows subsequent prediction of HHF risk for subjects in this study to observe the changes to their risk level. The criteria used to obtain the sample size for this study, while strict, also ensures completeness of the data used for analysis. Furthermore, HHF records were not available for certain institutions after 2017 which indicates that the true incidence of HHF risk could potentially be higher.

## Conclusions

In conclusion, this paper uncovered distinct latent classes of HbA_1c_ trajectories and analysed its relation to survival probability of a first HHF based on a group of Southeast Asian population. Generally, a low and stable HbA_1c_ trajectory or stricter glycaemic control with minimal variability leads to better survival prognosis as compared to high and unstable trajectories. The importance of defining an appropriate baseline cannot be overstated, as it has the ability to alter class memberships of pre-existing and new subjects. Nonetheless, this clustering framework has the potential to allow the identification of patients who face higher risks in developing heart failure leading to a need for hospitalization, allowing clinicians to efficiently direct resources for a more strategic approach in managing T2DM for each subgroup in order to reduce risks of cardiovascular comorbidities.
